# Recalibrating Everyday Futures during the COVID-19 Pandemic: Futures Fissured, on Standby and Reset in Mass Observation Responses

**DOI:** 10.1177/00380385231156651

**Published:** 2023-04-25

**Authors:** Rebecca Coleman, Dawn Lyon

**Affiliations:** University of Bristol, UK; University of Kent, UK

**Keywords:** everyday life, futures, mass observation, temporality, time

## Abstract

This article contributes to sociologies of futures by arguing that quotidian imaginations, makings and experiences of futures are crucial to social life. We develop Sharma’s concept of recalibration to understand ongoing and multiple adjustments of present–future relations, focusing on how these were articulated by Mass Observation writers in the UK during the early part of the COVID-19 pandemic. We identify three key modes of recalibration: *fissure*, where a break between the present and future means the future is difficult to imagine; *standby*, where the present is expanded but there is an alertness to the future, and; *reset*, where futures are modestly and radically recalibrated through a post-pandemic imaginary. We argue for sociologies of futures that can account for the diverse and contradictory ways in which futures emerge from and compose everyday life at different scales.

## Introduction

Recent sociological attention has focused on the role of the future in organising, shaping and governing social worlds, especially at a macro-social level (Adam and Groves, 2007). The future has thus become an ‘analytic object’ (Brown and Michael, 2003: 4) that requires scrutiny and exploration (Beckert, 2013; Coleman and Tutton, 2017; Levitas, 1993). In this article, we bring together research on futures, temporality and everyday life to contribute to and extend sociologies of futures, arguing that quotidian imaginations and makings of futures are little studied but crucial aspects of social life and vital for sociological understandings of futures. In particular, we take up time studies scholar Sharma’s (2014: 18) concept of ‘recalibration’ to understand ‘the multiple ways in which individuals and social groups synchronise their body clocks, their senses of the future or the present, to an exterior relation – be that another person, pace, technology, chronometer, institution, or ideology’. We put this in dialogue with ‘futures studies’ work, which recognises ongoing temporal adjustments at different scales from the mundane to life trajectories (Mische, 2009; Tavory and Eliasoph, 2013) as well as that which emphasises a dominant contemporary temporal regime of anticipation, whereby the future is anticipated in, and actively shapes, the present (Adams et al., 2009, Anderson, 2010). We argue that while the recalibration of present–future relations is a persistent and taken-for-granted part of everyday life, it was made apparent through the COVID-19 pandemic. The pandemic might therefore be understood as a ‘site of hyperprojectivity’ (Mische, 2014: 438), enabling people to reflect on their plural and shifting temporal experiences and begin to make sense of how present–future relations are shaped, and sociologists to develop new conceptions of the making of futures.

We present research undertaken in the UK with Mass Observation Project (MO) during the pandemic about people’s everyday experiences of presents and futures. We examine how futures were articulated in written reflective responses submitted to a 2020 MO directive we co-commissioned on COVID-19 and time. Building on arguments that MO ‘offer[s] detailed and exceptionally rich accounts of the fibre of everyday life’ (Casey et al., 2014: 1.1), we see the responses as providing valuable insights into how futures are imagined, organised and experienced through everyday practices during a period of enormous social upheaval. We focus on how MO panellists highlighted the recalibration of their relations between the present and future and identify three key modes. The first, *fissure*, conveys a break between the present and future. Futures become difficult to imagine and present–future relations are recalibrated as versions of the future previously anticipated disappear, radically altering experiences of the present. Second, *standby* evokes a situation of ‘in|activity’ (Kemmer et al., 2021), where vitality is held in check. We explore this through an attention to the recalibration of present–future relations where there is an alertness to the future in an extended present (Nowotny, 1994). Third, *reset* refers to a post-pandemic imaginary where futures are recalibrated in modest and radical ways. While these modes emerged from an analysis of everyday experiences of the COVID-19 pandemic, they offer a framework for present–future relations for other contexts where instability and uncertainty loom large, and for sociological conceptual understanding of these temporal relationships more generally.

## Recalibration and Sociological Understandings of Futures

Sharma introduced the concept of ‘recalibration’ in her ground-breaking work on the politics of time. She sought to add texture and complexity to the elision of speed with modernity from the classical accounts of Marx and Simmel to recent renewed sociological attention to social acceleration (Rosa, 2013), alongside the felt experience of acceleration in everyday life (Wajcman, 2015). Through ethnographic research with people whose jobs require specific attention to time (taxi-drivers, frequent-flyer business travellers, corporate yoga instructors), she details how speed is differentially experienced and made through ongoing adjustments, or the recalibration, of social relations. The taxi-driver, for example, often incurs long waits to ensure they are ready to respond to the needs of the business traveller, and is then required to move quickly to deliver the business traveller to their appointment.

Sharma’s (2014) conceptual framework and analysis highlights the significance of temporality to power relations; indeed, she defines temporality as ‘a specific experience of time that is structured in specific political and economic contexts’ (2014: 9). Thus, while ‘time’ refers to generalised and standardised units (e.g. clock time), or to specific historical periods, temporality is ‘*lived time*’ (Sharma, 2014: 9, emphasis in original), or what Southerton (2020: 5) terms, ‘perceptions of social phenomena related to or of time’. We build on these definitions of temporality to attend to the MO writers’ everyday lived experiences during the pandemic and, more specifically, to the shifting relations between presents and futures. We see the concept of recalibration as a productive way to understand these moving temporal relations. Uneven, fluctuating and open-ended processes whereby ‘the invitations and expectations to recalibrate time [. . .] permeate the social fabric differently for distinctive populations’ mean that social groups must ‘learn how to deal with time, be on top of one’s time, [. . .] learn when to be fast and when to be slow’ (Sharma, 2014: 18). The term *re*calibration indicates that temporal relations must be calibrated again and again. We build on Sharma’s concept of recalibration in our analysis of the MO responses, valuing an approach that empirically examines the lived experience of time, and in this article specifically present–future relations. While Sharma notes that recalibration aims to understand how individual and social groups adapt ‘their senses of the future or the present, to an exterior relation’ (Sharma, 2014: 18), this aspect of her argument is less developed than a general concern with time and temporality. We therefore extend the scope of the concept of recalibration through our analysis of present–future relations as they were reflected on by MO panellists.

To develop the concept of recalibration, we make connections with existing sociological research, which offers a range of frameworks for understanding the future. Mische (2009: 696) proposes ‘projectivity’ as a neglected aspect of how ‘human action is constructed within an imaginative horizon of multiple plans and possibilities’. She argues for an ‘open, indeterminate, “polythetic” perception of the field from the point of view of the actor surveying the future’ rather than the more ‘monothetic’ view captured in retrospective interpretation (Mische, 2009: 697). She offers a rich set of cognitive dimensions of future projections: reach, breadth, clarity, contingency, expandability, volition, sociality, connectivity and genre, which provide ‘a cognate set of categories with which to think’ (Wagner-Pacifici, 2009: 706). Working with these dimensions in empirical analysis surfaces aspects of projectivity that can easily be missed, and we draw on several of them below. However, despite the emphasis on contingency in Mische’s work, there is an inherent linearity of present–future relations that assume an agent ‘projecting’ into a future that is assured, if changing. What we argue is evident in the MO responses is a decidedly uncertain future that projects into and shapes the present, complicating linear understandings of the relations between presents and futures and drawing attention to the processes of recalibration that this implies.

In a further framework, Tavory and Eliasoph (2013) argue that ‘projects’ emphasise individual volition and conceptualise ‘coordinated futures’ arising from interaction with others. They propose three modes of future coordination that operate autonomously as well as both in concert and at odds with one another. ‘Protention’ refers to moment-by-moment anticipations; ‘trajectories’ are the culturally shaped paths (which take the form of projects and narratives) that people deliberately follow within larger timeframes than the immediate future; and ‘plans and temporal landscapes’ are the ‘naturalized [. . .] bedrock on which other future-oriented trajectories are being performed’ (Tavory and Eliasoph, 2013: 916). A key element of future-making here is (following Bourdieu) the ‘feel’ for the future that informs people’s actions and directions. We argue that the pandemic unsettled taken-for-granted senses of a future, hence the emphasis we place on processes of recalibration in our analysis. It allows us to capture the granularity of adjustments as present–future relations are remade. We nevertheless draw on Tavory and Eliasoph’s notion of ‘temporal landscapes’ in our analysis to explore this shift and make use of ‘protentions’ in our discussion of the im/possibility of imagining a future during COVID-19.

Other social scientific research on futures examines anticipation as an organising principle of social life in the contemporary Global North. Poli (2017: 3) defines anticipatory behaviour and systems as ‘tak[ing] decisions in the present according to “anticipations” about something that may eventually happen in the future’. Adams et al. (2009: 247) argue that ‘[a]nticipation is the palpable effect of the speculative future on the present’, where ‘the future sets the conditions of possibility for action in the present, in which the future is inhabited in the present’ (2009: 249). Anderson (2010) identifies precaution, pre-emption and preparedness as logics that attempt to intervene, in the present, in an anticipated future. For him, anticipatory practices involve calculating, imagining and performing the future, which is ‘folded into the here and now’ (Anderson, 2010: 778). The uncertainty of the future ‘becomes cause and justification for some form of action in the here and now’, prompting a ‘proliferation of anticipatory action’ (Anderson, 2010: 778). What is clear here is the non-linearity of time, whereby the future is not that which the present unspools into, but is also that which acts on – ‘palpabl[y] effect[s]’ – the present.

The concept of recalibration enables us to attend to three interconnected aspects of present–future relations during the COVID-19 pandemic. First, it enables a focus on the significance of futures to everyday life in the present, what Adam and Groves (2007: 123) term the lived future; ‘the future experienced as a constitutive element of the present’. Here, we understand everyday life as what Neal and Murji (2015: 812) explain as both ‘the mundane, the routines in (and of) social relations’ and as ‘characterised by ambivalences, perils, puzzles, contradictions, accommodations and transformative possibilities’. Second, it enables us to think across the different scales of everyday life, recognising that ‘micro social life, the banal and the familiar are co-constitutive of the wider complexities, structures and processes of historical and contemporary social worlds’ (Neal and Murji, 2015: 812). Helpful here are Michael’s (2017: 510) definitions of ‘Big Futures’, concerned with significant novel technological developments, and ‘Little Futures’, which ‘entail much smaller, more circumscribed changes, whose impact describes a relatively tighter spatio-temporal horizon’, notably ‘the local unfolding of everyday life’. If Big Futures ‘imply very substantial, qualitative changes (e.g. some sort of epochal “break”), that are widespread and far-reaching, whose spatio-temporal horizons are relatively large-scale’ (Michael, 2017: 510), Michael (2017: 511) argues that Little Futures and ‘everyday life might, nevertheless, be a source – rather than simply a critical illustration – of Big Futures’. Third, it enables us to explore the recursiveness of present–future relations, and to consider how futures act on, organise and pattern everyday presents. Overall, working with the concept of recalibration facilitates an exploration of the processes of adjustment that arise from and produce new temporal relations in which futures – or their absence – are central.

## Studying Everyday Futures through a Mass Observation Directive

Our approach to studying ‘lived futures’ (Adam and Groves, 2007) in the context of the COVID-19 pandemic was developed through collaboration with MO, which has an important role in ‘recording everyday life in Britain’ (https://www.massobs.org.uk/, Adkins, 2017; Casey et al., 2014; Hubble, 2006; Savage, 2007). MO has ‘played witness to the great and the small events of everyday life’ (Casey et al., 2014: 1.1), encouraging writers ‘to produce thickly rendered accounts of the present, and to explain the taken-for-granted aspects of it’ (Highmore, 2010: 128). We see the responses as ‘collective or multiple autobiography’ (Sheridan, 1993: 34 in Kramer, 2014: 4.5), and dynamic, varied and inconsistent and therefore offering a multiplicity of views (Highmore, 2010). We approached MO to study time and the pandemic in an ethically appropriate manner; MO panellists would expect to receive a directive and are well versed in responding (or not) to prompts on specific aspects of daily life, and so we felt we would not be imposing on people at a potentially difficult time. We also sought to contribute to MO’s public accessibility, ensuring responses to the directive were a potential future resource for researchers.

With colleagues,^1^ we commissioned a MO directive, asking writers to reflect on how time was made, experienced and imagined during the first year of the pandemic, when people’s awareness of the changing experience of time was heightened. In particular, we asked them to:

tell us how time in your daily lives may have changed – or stayed the same – as a result of the pandemic [. . .] look back on your experiences of time and COVID-19 so far, [and] consider what it means to you for life to get ‘back to normal’ (if it is) in the present day, and imagine what you think the future might hold.

There were specific prompts on rhythms and routine, homelife, media and technology and waiting and we explicitly asked people to reflect on their imaginations of the future as well as on their everyday presents (for the full directive see: http://www.massobs.org.uk/images/Summer_Directive2020_FINAL.pdf).

The directive was sent to 758 people (an unusually high number as during the pandemic the panel swelled from 450–500 with people signing up for activities they could do from home)^2^ in August 2020 for submission to MO until the end of November 2020. At this point of the pandemic, England, Wales, Scotland and Northern Ireland had variously relaxed and reinstated COVID-19 restrictions following their first introduction on 26 March 2020, a day after the Coronavirus Act (2020) was passed. Amid much public discourse about ‘the return to normal’, a second wave of the virus was nevertheless anticipated and further ‘lockdowns’ were introduced throughout winter 2020/2021. Vaccines were being trialled so were ‘on the horizon’ but their effectiveness was not known until December 2020.

We received electronic copies of 228 submissions (30% response rate) ranging from a few lines to eight pages in length. They were submitted by 161 (70.6%) respondents identifying as women, 49 (21.5%) as men, two as non-binary and 16 where gender identity is unknown. While the age categories of 60–69 and 70–79 each contain more respondents than other age groups, 46.1% of the sample where age is declared is under the age of 60. In terms of labour market participation, the sample was evenly split between those in paid work (or seeking work) and those who were retired (45.6% vs 46.1%, the rest either a homemaker, 2.2%, student, 3.1% or unknown, 3.1%), figures that align closely to age with less than 5% of the sample in work being 60 and over. Occupational status suggests the sample is dominated by middle-class writers with 22.3% of those where details are known in or previously in higher managerial/professional positions, 19.1% in intermediate occupations and 28.6% in lower managerial/professional positions, amounting to two-thirds of the writers. Where location is known, 27.4% of the writers are based in London or the South-East with a further 12.9% living elsewhere in the South; 31.8% based in the Midlands and 16.4% in the North-West or North-East. Those in Scotland, Wales and Northern Ireland combined represent a further 10.0% and several are resident abroad (excluded from this analysis).

We share details of the respondents for transparency but our research does not seek to provide a representative sample of the UK population, nor does our analysis systematically compare experiences of different groups according to their socio-economic characteristics, especially since they are incomplete, notably a lack of data on ethnicity; they are far from representative; and are internally heterogenous. Instead, we draw on them to conceptualise different sets of present–future relations that have broader theoretical and empirical salience. Our analysis is organised around three key modes in which the future is recalibrated in relation to the present: fissure; standby; reset. These modes emerged from our immersion in the data, coding in the qualitative software program, NVivo (version 1.7.1), and thematic analysis drawing on dimensions of the existing conceptual frameworks we discuss above. They are not intended as a comprehensive ‘typology’ of futures during the COVID-19 pandemic, as we acknowledge the specificity of the data we examine, that there is a plurality of futures in everyday life beyond those we discuss (Michael, 2017) and that within each mode there are divergences as well as coherence. Neither do we intend our conceptualisation of future–present relations as a chronological progression from ‘no future’ (fissure), to an absorption in the present (standby), to a re-imagining of futures (reset) as these relations were articulated during the same time period, sometimes by the same writer. Fissure, standby and resets, then, exist alongside and overlap with each other and collectively offer insights into the recalibration of present–future relations.

## Fissure: The Impossibility of Imagining a Future

There has been much discussion of ‘no future’, or what Adam and Groves (2007: 10) call ‘empty futures’, from theorists concerned with sexuality (Edelman, 2011), digital capitalism (Beradi, 2011), debt (Lazzarato, 2011), the climate crisis (Urry, 2011) and nuclear weapons (Tutton, this issue). There is less attention paid to the sudden or slow process of ‘recalibration’ from a situation in which everyday futures are nevertheless envisaged to one where ordinary futures are erased – such as that arising from the COVID-19 pandemic. In this section, we work with the notion of fissure as a temporal opening, fracture or a crack, rather than an all-encompassing clean break. We deploy it to recognise how for many writers, the future was difficult, if not impossible, to imagine and how this meant a recalibration of present–future relations. Fissure draws attention to the contradiction of being un/able to imagine futures. Even when it is unimaginable or apparently absent, the future plays a significant role in everyday life in the present; fissure captures how present–future relations are dynamic and recursive rather than always linear and progressive.

When writing about her imagination of the future, S1399 states: ‘Future – absolutely no idea’, and later: ‘I just can’t think of what the FUTURE might be like’ (S1399, Female, 71, White, Kent, Married). S1399’s blankness about the future draws attention to the challenge of recalibration in the face of such profound disruption, and is expressed by other writers, who discuss how the cancellation of plans changed their sense of themselves and of time more generally. For example, L7173 writes, ‘My life before [the COVID-19 pandemic] was always planning the next event, looking forward. Suddenly, everything in the foreseeable was cancelled. I was unable to travel on public transport and found myself confined to my village’ (L7173, No Information). A number of writers wrote about how plans for holidays and birthday, wedding and anniversary celebrations were postponed and/or cancelled, with some noting that they were anticipating further postponement or cancellation due to an impending second wave of the virus. Future perspectives and practices at different registers – protentions, trajectories and landscapes (Tavory and Eliasoph, 2013) – were all unsettled.

The cancellation of plans and the recalibration of the quotidian generates for some MO writers an uncertain and difficult to experience present. Fissure conveys a sense of the cracks in the everyday as well as in present–future connections. P7411 writes that, ‘[t]here is nothing to look forward to any more as nothing is certain any more’ (P7411, Female, 58, Staffordshire, Carer, Married). Such recalibrations create a range of feelings:

My experience of time has radically changed recently. I try not to look forward anymore because disappointment is so hard to bear. I have become slightly agoraphobic about going out. (P7535, Female, 78, Oxfordshire, Retired Teacher and Welfare Officer, Widow)

As time goes by there is obviously no end in sight or any expectation that life can resume as normal and the lack of a vision of the future just makes me all the more depressed. (K7114, Female, 77, Derbyshire, Retired Researcher, Widow)

These examples indicate how not being able to ‘look forward’ unsettles ‘normal’ life and produces an uncertain present experienced in terms of depression, disappointment and agoraphobia, and a more general dissipation of hope:

When it comes to thinking of future time, for example looking forward to something, my mind has nothing to work on. All is uncertain. [. . .]. I have always had an instinctive feeling of hope – that something would turn up, that solutions to problems would occur to me, new opportunities would appear. (S6385, Male, 78, Derbyshire, Retired Town Planner, Widower)

The possibility of ‘no future’ feeds into and shapes the present as uncertain. These reflections on the recalibration of present–future relations indicate how important the future is to people’s everyday lives and sense of what it means to inhabit the world. Whether the ‘reach’ (Mische, 2009) of the future is distant or proximate, it provides a sense of what, in Berlant’s (2011: 24) terms, it is to ‘keep on living on and to look forward to being in the world’, and of something that provides life with meaning and pleasure.

Lost futures also lead to a fissure in the present when writers reflect on their time being ‘permanently taken away’: ‘My parents are old and so it does feel like we may not have much more time together, that the lockdown is permanently taking time away from us that we won’t get back’ (W6757, No Information). Here present–future relations are recalibrated both in terms of the uncertainty of the future and because the future is literally diminished; this time cannot be recovered. Other writers use the language of time being ‘stolen’. H5845 noted she ‘feel[s] a little bit like the government are robbing me of my life’ (H5845, Female, 40, Nottingham, Midwife, Married) and H1745 reflects on her 95-year-old mother telling ‘everyone she “resented” the way in which Covid had robbed her of so much time when she presumably has a limited time left on Earth’ (H1745, Female, 69, London, Researcher/Writer/Editor, Widow/Living with Partner).

The fissure at the prospect of ‘no future’ indicates that the present does not (only) lead onto the future, but that the future (also) loops into the present, a recursive reflection that intensifies the present. In Mische’s (2009) terms its ‘expandability’ is contracting. In the theories of anticipation introduced above, the non-linearity of presents/futures has been studied through a focus on the future as an anticipated threat (e.g. acts of terrorism, a disaster, an emergency). In this sense, the future is in the present because the future is secure; it exists, can be imagined or anticipated, and must be mobilised around ‘as if the future is what matters most’ (Adams et al., 2009: 248). The present–future relations we explore through fissure, however, emphasise the experience of living without a sense of the future, and/or with a curtailed future, and the recalibration that this sudden change entails as lockdown and other pandemic measures took hold. For the MO panellists discussed so far, the future is in the present because it cannot be imagined or anticipated. The unimaginable future creates the uncertain present. The future shapes the present as a result of its apparent absence. The anticipated future threat has happened – the pandemic is here and now, in the present – and MO panellists are grappling with newly recalibrated temporal relations.

## Standby: Suspended Futures, Alert Presents

Standby as we deploy it here conceptualises a sense of possibility and vitality on the one hand and the experience of restraint and stoppage on the other. Kemmer et al. (2021: 1), define standby ‘as a state of “in|activity” that indicates readiness without immediate engagement, but that nevertheless requires and generates energy, resources, and relations’. Taking up this condition of in|activity as key to standby, we consider its temporal relations in terms of the present–future. While fissure explores the sense of no future and resultant recursive intensification of the present, the recalibration of the present–future relations of standby suggests a form of limbo that reverberates both with possibility and the frustration of restrictions. Standby for us indicates the contradictory and confusing condition of a paused present where the future is both uncertain and is capable of being reactivated.

The uncertainty and continued inability to look forward is described by some of the writers in terms of being ‘in limbo’ and ‘waiting for things to end or change, for this all to be over’ as K7050 put it (K7050, Male, 36, Surrey, Civil Servant). In these senses, as another writes, ‘Life does seem to be “on hold”, and quite stuck. It is harder as the pandemic goes on to feel that an end is in sight’ (K7066, Female, 49, London, Librarian, In a Relationship). B7515 writes how ‘recently I find that I just want to hunker down and wait for it all to pass, which feels rather defeatist, but it is quite hard to stay motivated and keep going, I think’ (B7517, Female, 56, Greater Manchester, Digital Forensic Investigator, Married) while H7512 notes that ‘[t]o me it feels like we are trapped on a hamster wheel and it is never ending’ (H7512, Female, 53, North-West, Retired Detective, Married to Another Woman, Separate Homes). These two extracts demonstrate how a shared temporal condition of life being ‘on hold’ can be experienced differently. For both, the future is contingent (Mische, 2009) on the unfolding regulation of everyday life. B7515 experiences a desire to ‘hunker down’ and a difficulty in ‘keep[ing] going’ and H7512 a ‘never ending’ cycle that demands she keeps moving.

We explicitly asked writers about their experience of waiting, and while reflections range from the concrete and mundane to the more diffuse, they evoke a sense of constrained energy. For example, two writers note they are waiting to be able to meet in person, with A6936 writing she is ‘champing at the bit to get back to the real thing!’ (A6936, Female, 69, Bristol, Retired Civil Servant, Widow) and F6959 writing, ‘I can’t wait until we’ll be able to banish online everything into the past and meet in person again’ (F6959, Female, 20, London, Student, Single). Waiting is not only a sense of stilled time but also, as Bissell (2007: 287) describes, gives rise to ‘an enlivened corporeal sensibility where bodies are highly attuned to their immediate environment and themselves’. ‘Standby’ suggests a ‘temporal modality’ (Deville, 2021) characterised by this dynamic experience of waiting in a paused present.

This quality of being ‘on hold’ means that in Mische’s terms, the future has little ‘reach’ beyond the day to day. There is a collective experience – ‘sociality’ following Mische – of not being able to count on a particular future and a widespread sense of ‘contingency’ in relation to a new wave of the pandemic. B3635 discusses how:

[m]y experiences of putting my life on hold included having to cancel my wedding and honeymoon. [. . .] The honeymoon was cancelled a few weeks later and hopefully it, along with the wedding, will go ahead next year and there won’t be another wave of the virus to postpone everything again. (B3635, Female, 44, Essex, Homemaker, Engaged)

T5672 writes how ‘[w]e haven’t had a holiday this year and don’t expect to. We had something booked for Christmas which has now been cancelled, and I wouldn’t be too surprised if we don’t have a holiday in 2021 either’ (T5672, Male, 37, Bristol, Senior Manager). The sense of being pulled into temporal relations at different scales is powerful in the MO submissions as standby is ‘a field of tension between the contingent and persistent, between the exceptional and ordinary’ (Kemmer et al., 2021: 1). As B6659 writes:

There is so much unknown. I keep in mind that full lock-down might occur again. I try to be optimistic, and make plans. But I try to remain realistic – that all plans may be abandoned. I have scrap booked all my cancelled tickets for this year. But I have optimistically purchased a scrapbook for next year. (B6659, Female, 67, East Yorkshire, Retired University Lecturer, Widow)

Here, there is reflection on the simultaneity of a cancelled future and a future that is imaginable, as plans are made with the recognition that they may be ‘abandoned’, and a future that is remembered. In terms of standby as a state of in|activity, B6659’s reflections convey a sense of to and fro and the energy and activity that encompasses the present when the future can be imagined but is not guaranteed. Similarly, C3210 writes:

The uncertainty and not knowing how long the wait for ‘normality’ will be is the hardest part of this for me. It’s difficult to look forward to things in the future because we have no way of knowing whether they’ll happen. [. . .] Usually I see August bank holiday as a real checkpoint in the year; summer is all but over and I’d be looking forward to my favourite bit of the year, especially now I have a child. I love going out trick or treating with my son on Hallowe’en. Bonfire night has always been a favourite of mine and although I find Christmas stressful, I do enjoy the run up – that festive feeling, the food, the time off. But this year, who knows which bits of Autumn will happen. (C3210, 40, Female, Hertfordshire, Civil Partnered with Five-Year-Old Son)

Whereas with fissure the future acts on the present through its absence, here collective and public markers of the year that C3210 has been able to take for granted in the past are uncertain. K7114 notes how, ‘[a]s time goes by there is obviously no end in sight or any expectation that life can resume as normal and the lack of a vision of the future just makes me all the more depressed’ (K7114, Female, 77, Derbyshire, Retired Researcher, Widow). Evident in these accounts is a ‘craving for certainty [which] finds expression in the desire to know which step to take next and which direction to trust when all that surrounds one is the fog of uncharted directions’ (Nowotny, 2016: 14). It is not so much that the future does not exist, but more that the normal ability to ‘look forward’ is placed into question to produce what Nowotny calls an ‘extended present’, which:

tries to diminish the uncertainty of the future by recalling cyclicality and seeking to combine it with linearity. The present is no longer interpreted merely as a part of the way on the straight line leading to the future open to progress, but as part of a cyclical movement. (Nowotny, 1994: 58)

This cyclicality is neatly expressed in the image of the ‘hamster wheel’ that H7512 mentions above, and it also encompasses the more general experience of standby where the present is full of activity and seems to not move on.

Others articulate their experience of waiting in terms of specific restrictions on seeing family and friends, highlighting in particular the care involved in their decision making. C5991 runs through a list of people she would like to see – including her mum, grandchildren, daughter, friend, and neighbour – and writes, ‘I can’t do any of these things until I have either had the vaccine or decided I don’t care whether Covid kills me and/or my husband’ (C5991, Female, 66, Essex, Retired Office Worker, Married). This extract highlights the threat of the virus as something that is both present – its presence requires these restrictions – and is anticipated as a possibility in the future – the worry that she or her husband might catch COVID-19 and be killed by it. The standby state that this writer is in involves an on-hold present at the same time as what Adams et al. (2009: 254) describe as an ‘alertness and vigilance [to the future] as normative affective states’; the kind of ‘readiness without immediate engagement’ that Kemmer et al. note when defining ‘standby’.

## Reset: Recalibration to a Different Future

We deploy the term reset to draw attention to another recalibration of present–future relations, in terms of confronting the future anew and reassessing what is important. Above we noted the ‘re’ of ‘recalibration’ emphasises that recalibration is ongoing. Here we highlight the ‘re’ of ‘reset’ to draw attention to how the prefix, ‘re’, refers to processes of creating anew, going back, being behind or after as well as repeating (Coleman, 2020). Resets, then, involve multiple temporal relations, including continuities and changes and can be more or less spectacular, echoing Augé’s (2015) distinction between the future as succession and as inauguration, continuity or rupture prompting a new beginning. Indeed, for some writers, the pandemic produces a ‘minor ripple’, whereas for others it is a ‘turning point’ (Abbott, 2001) where there is a fundamental recalibration of what their future should hold and a concrete making of these futures within the present. In the present–future relation we call reset, and in contrast to fissure and standby, it becomes possible to imagine the future as different from the present. The ‘temporal landscape’ (Tavory and Eliasoph, 2013) has been made evident and this has created a space for it to be reset.

First, we consider writers for whom resets are ordinary and tentative. These cases can be understood in terms of the ‘return to normal’ much heralded during the pandemic, where ‘the normal’ refers to the mundane and, until lockdown, often taken-for-granted aspects of everyday life. The ‘reach’ of the future in Mische’s (2009) terms is both a reach back to previous patterns of everyday life and a reach into the near future in which they might be re-established – although there is considerable uncertainty here. Some writers focus on ‘resets’ in everyday routine, for instance, how they would like to be able to continue to work from home, noting reasons of well-being and flexibility – ‘I’m not so tired, and I prefer the extra flexibility as to when I do my work’ (W6757, No Information) – and extra time to spend with family – ‘I hope we continue to be able to spend more of the before and after school time with the kids’ (T5672, Male, 37, Bristol, Senior Manager, Married). In such cases it is that resets have drawn attention to what matters in the mundane and ordinary aspects of everyday life. As H5845 notes, ‘What the pandemic has taught me is that we must not take anything, however big or small for granted as it can change in a heartbeat’ (H5845, Female, 40, Nottinghamshire, Midwife, Married).

For some, the pandemic felt like ‘it was a gift’ from which the future might be approached differently. A5854 writes,

There was part of me that had longed for days with no social events, no journeys etc. To be able to have ‘empty days’. [. . .] time to focus on things I wanted and needed to do and hadn’t found time to do. We are blessed with being retired on good pensions, having a garden and small orchard and in good health and very good neighbours. (A5854, Female, 75, Somerset, Artist, Married)

Here, a reset involved the evaluation of what everyday life might involve when longed-for ‘empty days’ become the norm. B5189 reflected on how the lockdown had also enabled her to assess things she did not like doing and would not do again:

I’ve never liked meeting people in cafes, seems like a huge waste of money to me and now I no longer do it and probably won’t again. My pension gives me £7.5k a year, people think they are being kind by inviting me for lunch or dinner ‘I’ll treat you’ they say. It’s really not the pleasure they think it is for me. The food is never as good, fresh, organic or homegrown as my food. I don’t ever want to go into a cafe or restaurant again. I have learnt how to say no thank you over this period. (B5189, Female, 71, Lancashire, Yoga Teacher, Single)

Others noted the new habits they had been able to establish, reflecting on how the pandemic had enabled change, or the ability to ‘reset my life’:

By the middle of May, I realised that I had put so much weight on during Lockdown that I had to do something about it. I signed up for Weight Watchers and started jogging in the park. I have now lost the weight I gained, plus another 9lbs, and have started running about five times a week. I developed a new exercise routine and have managed to fit it in around work, now that I’m off furlough. This is an incredibly positive result of Lockdown. I was able to reset my life and resume it in a healthier direction. (G7105, Female, 50, Somerset, Invoice Clerk, Divorced)

Here, the reset is neither an abrupt nor singular moment (Abbott, 2001: 247) as the initiation of a new exercise routine during the pandemic is continued once the diarist is back at work. This reset, then, provides a continuity between lockdown and what the writer imagines as their post-pandemic life.

Second, we consider more radical resets. For Abbott (2001: 244), in contrast to ‘minor ripples’ or ‘random episodes’, ‘turning points’ interrupt or ‘jolt’ people ‘into and out of trajectories’, chiming with Tavory and Eliasoph’s conceptualisation of trajectories discussed above. While it is usually in retrospect that the extent of change can be grasped, some of the authors write with conviction about the difference that the pandemic has already made in the patterns of their everyday lives and the new directions they have taken. For example, some respondents wrote about deciding to make significant breaks in how they were living and a refusal to return to the previous practices. A6936 reflects on an impending house move:

The most significant thing about lockdown to me was the realisation that I didn’t want to grow old in the home that I have lived in for 38 years, and now would be the time to move. [. . .] Of course during lockdown I hated being ‘confined to barracks’ and it forced me to think about the issues that I had been avoiding for years. [I] put my house on the market. It sold in 5 weeks and I have chosen a flat, so my homelife in the future will be elsewhere, when the move goes through. (A6936, Female, 69, Bristol, Retired Civil Servant, Widow)

G6209 writes about an impending relationship separation:

Having genuinely considered separating from my husband for a number of years – more so since he semi-retired and became a Consultant mainly working from home – I now know that this is something I definitely want to do. We have been together for 27 years and married for 19 and that’s quite enough now. We have classically ‘drifted apart’ and we now need our own spaces. (G6209, No Information)

In both of these cases, resets are fundamental to everyday life as the future is recalibrated in a different form to the present. For E5559 the future is imagined in terms of a desire to minimally affect himself and the environment:

As lockdown is slowly lifted, I realise that I don’t want to go back to the way things were. In the next few months I plan to leave my job, and to try and ﬁnd a way to survive that doesn’t harm me, the environment, and anything or anyone else. (E5559, Male, 53, Devon, Retail, Single)

The recalibration of present/future relations brought about by the pandemic is an opening to imagine futures that might be different from the present in which new possibilities emerge. The pandemic may come to be seen as the ongoing event that made change possible, or one that surfaced existing desires, most succinctly put by B6900 who writes,

Returning to the strangeness of this last year, I do think that for many, time is no longer something to take for granted and many people are now starting to look at the world around them and their time on earth a little differently than before. (B6900, No Information)

What this future might be clearly differs in terms of content, scale and scope but a desire to reset present–temporal relations is a strong theme in many accounts.

## Conclusion

In this article, we contribute to sociologies of the future by developing Sharma’s notion of recalibration to explore experiences of futures in everyday life. The making of ‘Little’ futures and their vital relations with wider social change is easily missed by sociological and allied research that focuses on time as epochal and macro shifts. However, by drawing on social scientific work on anticipation, projectivity and temporal relations, we argue that a distinctive sociological understanding of futures attends to the everyday and recognises the co-constitution of ‘Little’ and ‘Big’ futures (Michael, 2017). More specifically, we highlight the significance of everyday present–future relations as they emerge during the early phases of the pandemic in the MO responses. Recalibration offers a novel conceptual approach to grasp present–future connections and attune to subtle ways in which these different temporal relations are articulated during the pandemic and beyond.

Our analysis has identified three key modes of ‘lived futures’ (Adam and Groves, 2007) through which present–future relations are intimated – fissure, standby and reset. ‘Fissure’ captures the experience of no future arising from the apparent break between the present and future. The future, in its absence, shapes present experience and without a temporal horizon that includes the future, the present is itself jeopardised. With ‘standby’, the future is alive if difficult to enact, giving rise to an energetic experience of the present with a simultaneous readiness for the future starting up again. The experience of being ‘on hold’ or ‘suspended’ is not so much a clearly defined break or a chronological stage of being ‘in between’ the impossibility of imagining the future and being able to again, but is an experience of the recalibration of present–future relations in which the present comes to dominate. ‘Reset’ refers to an imaginary in which the post-pandemic future is recalibrated in more or less grand ways, drawing attention to the ruptures and continuities, ‘minor ripples’ and ‘turning points’ in present–future relations.

As stated at the outset, these modes are intended as analytically distinct rather than a typology in which individuals can be located. Instead, attending to ‘the complexity of lived time’ (Sharma, 2014: 6) involves identifying and exploring the multiplicity of temporal relations and how these co-exist. Across these modes, the concept of recalibration helps us understand how futures are constantly in the process of being imagined, experienced and made as we attend to ordinary processes of adjustment. Indeed, that recalibration is an ongoing and plural process is a key point in the approach we develop, as it highlights the unsettled character of present–future relations. Furthermore, recalibration allows us to attend to the non-linear relationships between presents and futures where the future is not (only) a not-yet time, but is felt and lived in the present. This recursiveness of present–future relations both builds on and troubles assumptions, including in existing sociological research, that the future is assured, even if contingent, and that time flows or is made by individuals linearly. While our analysis seeks to make sense of the distinctive period of the COVID-19 pandemic, it has broader implications. Indeed, we demonstrate and argue for sociologies of futures that account for the diverse and even contradictory ways in which futures both emerge from and compose everyday life at different scales.

## Notes

1. The directive was prepared collectively by colleagues engaged on our broader project, A Day at a Time: https://research.kent.ac.uk/daat-coronavirus/, which includes Simon Bailey, Michelle Bastian, Rebecca Coleman, Emily Grabham, Dawn Lyon and Dean Pierides.

2. Details from Jessica Scantlebury, MO Archivist, personal communication, 20 May 2022.
